# Gulliksen’s pool: A quick tool for preliminary detection of problematic items in item factor analysis

**DOI:** 10.1371/journal.pone.0290611

**Published:** 2023-08-25

**Authors:** Pere J. Ferrando, Urbano Lorenzo-Seva, M. Teresa Bargalló-Escrivà

**Affiliations:** Research Center for Behavioral Assessment, Universitat Rovira i Virgili, Tarragona, Spain; University of Castilla-La Mancha: Universidad de Castilla-La Mancha, SPAIN

## Abstract

Exploratory factor analysis is widely used for item analysis in the earlier stages of scale development, usually with large pools of items. In this scenario, the presence of inappropriate or ineffective items can hamper the process of analysis, making it very difficult to correctly assess dimensionality and structure. To minimize, this (quite frequent) problem, we propose and implement a simple procedure designed to flag potentially problematic items before we specify any particular factorial solution. The procedure defines regions of item appropriateness and efficiency based on the combined impact of two prior item features: extremeness and consistency. The general proposal is related to the most widely used frameworks for item analysis. The limits of the appropriateness regions are obtained by extensive simulation in conditions that mimic those found in applications. An Item Response Theory index of prior item efficiency is then defined, and a combined approach for selecting the most effective and problem-free item sub-set is proposed. The proposal is useful to normal-range measures, such as questionnaire surveys that elicit reports about non-extreme attitudes, facts, beliefs or states, or personality questionnaires that measure normal-range constructs. The procedure is implemented in a freeware software.

## 1. Introduction

Exploratory Factor Analysis (EFA) continues to be a very valuable tool in the development of measurement instruments in social science research, such as attitude measures, personality questionnaires or survey scales [1–3]. EFA is particularly useful in the earlier stages of the item analysis process, the process in which the items that will constitute the final version of the measure are selected on the basis of their contribution to the strength and meaning of the final structure of the instrument [4].

Some authors consider factor item analysis (FIA) as a global process in which alternative solutions are examined and the behavior of the items is assessed within each solution [5, 6]. While examining item properties in the context of a specific solution is indeed necessary, we believe that the selection process can be made more sequential and practical at its initial stages, in which large pools of items are usually analyzed [1, 3, 4, 7].

The authors of this article are developers of free software for exploratory FIA and also act as advisers for practitioners. In both roles, we have observed that most practical problems (a) occur in the earlier stages of the analysis, and (b) are due to the presence of problematic items that give rise to improper or distorted solutions which, in turn, make it very difficult to arrive at a correct dimensionality assessment and a clear structure. Thus, for example, a review of 320 queries showed that about 10% of them were related to non-positive definite correlation matrices, a situation which is frequently observed when problematic items are present in the dataset. This state of affairs is the impetus for the present developments.

The basic idea of our proposal is to develop a simple procedure that allows inappropriate and potentially problematic items to be flagged and eventually discarded at an early preliminary stage of FIA. So, the problems mentioned above will be avoided or, at least, greatly minimized. As for initial clarifications, first, the proposal is intended to be particularly useful for normal-range measures [8] and initial item pools that contain some flawed, poorly designed items. These conditions correspond to most of the queries we receive. However, as discussed below, the proposal is submitted to be also of interest in other scenarios. Second, the broad concept of “problematic” items would be used here to refer to items that can potentially lead to problems of (a) estimation (e.g. improper solutions), (b) biased estimates and distorted fit results, and (c) instability. On the other hand, for those items that produce insufficient (or less than expected) information, we will use the term “inefficient”.

So as to develop the basis idea above, we impose several requirements. First, the indices on which the proposal is based have to be part of (or can be naturally embedded within) the EFA model. Second, they must also be related to indices and procedures used in frameworks other than factor analysis (FA), specifically Classical Test Theory (CTT) and Item Response Theory (IRT). Finally, given their preliminary nature, the indices must be obtained without specifying or fitting any particular solution with a given number of factors.

From this starting point, the article aims to make several contributions. At the methodological level we propose a simple tool that enables the initial item pool to be inspected quickly so that the researcher can easily flag the potentially problematic items. The proposed regions of appropriateness are defined by extensive simulation, and the proposal takes into account: (a) the scenarios usually found in applications and (b) the sampling variability of the indices on which they are based. We also further propose a procedure for assessing prior item efficiency for those items that fall in the appropriateness region. Finally, we propose a combined approach in which the developments discussed here are related to previous existing proposals. At the instrumental level, the procedure is implemented in freeware software.

### 1.1. Background

G-Pool is based on item analysis developments made in three general domains: (a) CTT [9], (b) IRT [10], and (c) psychometric EFA [11].

Whether based on CTT or on IRT, the process of item analysis in test theory has mostly been based on unidimensional models, and the selection of items is intended to maximize measurement efficiency. So, under these frameworks, items are selected to allow the most accurate, informative and discriminating scores to be obtained [4].

The consensus position in test theory (both CTT or IRT) is that maximizing efficiency requires the two main item properties of extremeness (difficulty, location) and discriminating power to be assessed **jointly** [9, 12–15]. Based on this idea, a graphical representation of items as points in a bivariate plot, in which the abscissa was the item mean and the ordinate was the item-total correlation has been proposed ([9], p. 209). Henryson [13] explicitly proposed this plot as a method for item analysis (see also [15] for the case of ability items), and Mellenbergh [16] used it in the context of the FA model. In our previous research [17], we generalized the proposal to the case of graded-response or more continuous items. In all cases, however, the graphic device was only intended for unidimensional item pools.

A second test-theory contribution, also based on the efficiency concept, stems from a series of studies started by Brogden [18] and continued by Lord [19] and Cronbach [20] about what particular combinations of item locations and discriminations give rise to the most efficient and informative scores. Results suggested that, for the modest amount of discriminating power that items generally have, differentiation is maximum when all items are of medium extremeness. Summarizing previous research results, Henryson [13] suggested that, for binary items with average intercorrelations of .30 to .40, differentiation is maximum when the mean (p) item values range between .40 and .60.

The last test-theory contribution used here is the IRT concept of amount of item information. In most FA-related IRT models (see below) this amount increases as the item becomes less extreme and more discriminating, a result which agrees with the consensus position above. Within the IRT framework, however, many authors warn that a high amount of item discrimination (and, therefore, of information) is not necessarily a positive feature or an indicator of efficiency [21, 22]. These warnings are generally related to previous claims made by Cattell [5] and based on the concept of “bloated specifics’: that very high item discrimination or consistency is, in most cases, an indicator of item redundancy rather than of item efficiency [23]. In principle, this view suggests that there is an ‘optimal’ region of consistency/discrimination defined by intermediate values.

In contrast to CTT-based or IRT-based item analysis, EFA applications have (a) generally considered multidimensional solutions, and (b) been more concerned about the structural properties of the instrument than about the properties of the scores derived from its factor structure. More important, however, is that the analysis under this approach has been concerned far more with avoiding “problematic” items (that could distort the assessment of the dimensionality and structure of the instrument) than with maximizing the measurement efficiency that the non-problematic items provide.

### 1.2. Model description

Two versions of the general EFA model will be considered as bases for our proposal: (a) the linear model and (b) the nonlinear model [24]. In the first case, the item responses are treated as continuous-unbounded variables, and the general EFA model is fitted to the product-moment inter-item correlation matrix. Indices derived from the linear version are directly related to the usual CTT indices of extremeness and discrimination [13].

In nonlinear FA modeling, item responses are treated as ordered-categorical measures using an underlying-variables approach (UVA) in which the general EFA model is fitted to hypothetical, continuous response-strength variables that underlie the observed discrete item scores, which means that the EFA model is now fitted to the polychoric inter-item correlation matrix [25]. More important for the present purposes, however, is that with re-parametrization, when the UVA-EFA model is applied to binary item scores it becomes the IRT multidimensional two-parameter model, and, when applied to graded item scores, it becomes the multidimensional IRT graded response model [24].

**Fig 1:**
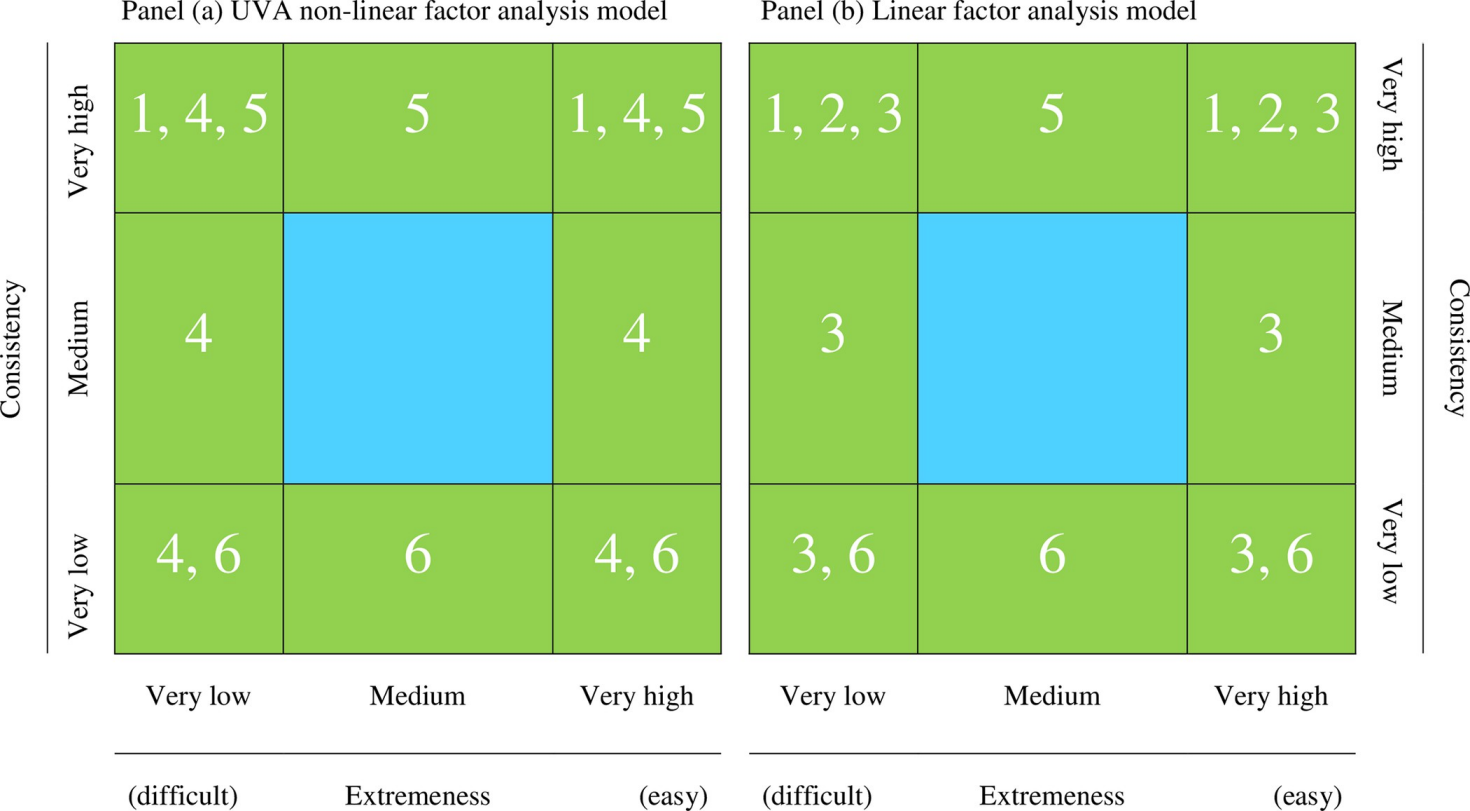
Gulliksen’s plot as related to potential problems for both factor models. Potential problems: 1. Improper solutions (NPD matrix, Heywood solution); 2. Incorrect goodness-of-fit assessment; 3. Biased parameter estimates; 4. Unstable parameter estimates; 5. Item redundancy; 6. Non-information, “noisy” items.

We turn now to the expected distortions when the two models discussed above are considered separately. They are summarized in Fig 1.

Panels (a) and (b) in Fig 1 provide (a) a preliminary graphic of regions defined by extremeness and consistency/discrimination, and (b) a list of the problems that are expected in the noncentral regions of the graphic. Note that most of the problems are similar in both the linear-FA and the UVA-FA models. So, the worst scenario is when items are both extreme and highly discriminating. However, under the linear FA model, this combination is expected to result in biased parameter estimates, whereas under the non-linear UVA-FA model, the expected result is not bias but mainly instability [26].

The problems caused by low-consistency items (bottom of the table) are discussed in detail in [27]. In addition to this discussion, the authors proposed to use an improved version of the “Measure of Sampling Adequacy” (MSA) [28] as a tool for detecting low-consistency, “noisy” items that behave almost at random. As discussed below, the MSA is a useful complement for the approach proposed here because it aims to detect items that do not “belong” to the domains defined by the remaining items in the set regardless of whether they can cause biases and/or estimation problems.

### 1.3. The basic proposal based on Gulliksen’s plot

The basic G-Pool proposal consists of (a) defining a “safety” central region of item appropriateness, like the one in Fig 1, and (b) flagging those items that fall outside the boundaries of this region as potentially problematic if they are included in further factorial solutions. In more detail, the items outside this region are those considered to be (a) potentially ineffective, (b) prone to leading to problems, or both. As discussed below, the boundaries of the appropriateness region will be determined by using extensive simulation.

Because the basic idea of G-pool is graphical, we shall describe its rationale in this way. It first requires a bivariate graphic to be constructed (see Fig 1) so that the index of item extremeness (*X* axis), and the index of item consistency/discrimination (*Y* axis) met the conditions discussed above. As for extremity, the original item location index in CTT (called the difficulty index) is the item mean computed on binary scores coded as 0 and 1 [29], and has an immediate interpretation as a proportion. Now, our proposal is to use the item mean as the index of item extremeness but always scaled in proportion-metric [4, 30]. So, regardless of the item response format, the index remains normed between 0 and 1, and (partly) retains the original proportion interpretation.

With regards to the consistency/discrimination measure, the best choice in our view is the square root of the prior communality estimate derived from Guttman’s image analysis, which we shall denote as *h*^*2*^ [31]. Thus, *h = sqrt* (*h*^*2*^) is the multiple correlation between the item (taken as a criterion) and the remaining items in the set. This estimate is a lower bound to the ‘true’ communality, and is obtained without having to specify a given number of factors (the basic requirement here). Furthermore, according to the redundancy-theory [32], *h*^*2*^ is not only a communality estimate, but also the redundancy index when the canonical correlation reduces to multiple regression. So, too high values of *sqrt* (*h*^*2*^) can be interpreted as indicators of item redundancy, as discussed above.

### 1.4. Sampling error

As some authors [13, 14] have already warned, indices of item extremeness and of item discrimination/consistency are both subject to sampling variations that should be taken into account when they are used for selection purposes. In terms of our proposal, sampling variation means that the points in the bivariate plot have a position that can fluctuate in the plane to some extent or another. There are two reasons why this variability needs to be taken into account. First, to appraise whether the sample on which the plot is based is large enough to guarantee stable estimates. And second, to develop an improved procedure for detecting the inappropriate items and prevent the results from being affected by capitalization on change. To address this issue, below we propose a robust procedure that (a) allows confidence intervals (CI) to be obtained for point estimates, and (b) minimizes the risk of capitalization on change by using a cross-validation assessment schema. In more detail, 95% of CI are obtained using bootstrap resampling, and items are flagged as problematic if both of the CI limits fall outside the appropriateness region. Furthermore, if the available sample is large enough, it can be split into two subsamples [33]. The first subsample can be used to decide which items are problematic, and the second sample to assess the extent to which the outcome of the selection is replicated. If the sample is not large enough, the researchers should collect a new sample in order to assess if the decisions taken in the initial sample can be replicated in a second sample.

### 1.5. Substantive considerations for using G-Pool

The proposal discussed so far is expected to be particularly useful when the conditions that are required for an appropriate functioning of the FIA model agree with the conditions that are desirable for the purposes and type of test that is analyzed. In general, these last conditions refer to normal-range measures, such as questionnaire surveys that elicit reports about non-extreme attitudes, facts, beliefs or states, or personality questionnaires that measure normal-range constructs. This type of measures seeks mostly items of moderate discrimination and medium extremeness, which coincides with the conditions our approach considers desirable. According to both, the literature review [3, 8] and our experience as advisers, these conditions correspond to most of the applications found in practice. In this scenario, the items that are flagged reflect generally design deficiencies, mainly, the use of redundant items, and/or the choice of inappropriate locations [4].

Other type of more specific measures, however, such as clinical inventories or selection tests might need items that are both extreme and highly discriminating [10, 34], which are precisely the sort of items that are more prone to giving problems when factor analyzed. In this case, it should become first clear that the items our procedure detects as potentially problematic are so regardless of the purpose and type of test. However, if the purpose requires the test to have a certain proportion of extreme and highly discriminating items, a trade off must be attained, and the researcher must carefully consider which items are to be maintained despite the problems that they may give rise to. In any case, obtaining information about which items are most likely to cause problems is always useful.

As for its intended use, finally, our proposal aims to flag ineffective and/or potentially problematic items, and takes care to avoid capitalization on chance by making it as robust as possible in this respect. However, it has purely technical bases and we do not recommend that it be used uncritically for automatically removing the flagged items. Rather, in our opinion, the practitioner should carefully inspect the item contents in order, at least, to understand why the item does not work. Indeed, as stated above, he/she might also decide that an item should be kept in spite of the warning the approach has given because the risk may be worth it for reasons beyond analysis convenience [35]. This issue is further discussed below.

## 2. Simulation study

### 2.1. Defining regions of appropriateness

In order to identify the regions that define the pool, we carried out a simulation study that took into account the item properties of extremeness (difficulty, location) and consistency. The background idea is that a scale should be defined by a minimum of five items [6]. Of these, four were generated as a set of medium items (i.e., they have medium extremeness/difficulties and consistency/discrimination power). The fifth item was the one whose extremeness and consistency/ discrimination power were manipulated. The parameter estimates related to this fifth item were closely inspected in order to decide whether the overall set of five items was appropriate. In addition, averaged bias and sampling error of the five items were also inspected. The procedure is explained now in detail.

### 2.2. Generating a set of items

As the items were generated to be ordinal, threshold response values were proposed so that the responses could be discretized. The threshold values were defined to manipulate ten levels of item extremeness. For example, for two response categories (i.e., binary items) the extremeness levels were the values shown in Table 1.

**Table 1:** Ten levels of threshold intervals used in the simulation study.

Level	Threshold interval
1	.00 –.09
2	.10 –.19
3	.20 –.29
4	.30 –.39
5	.40 –.49
6	.50 –.59
7	.60 –.69
8	.70 –.79
9	.80 –.89
10	.90 –.99

In order to generate an item with a given level of extremeness, the value was randomly chosen from the interval associated with the level at hand. For example, in order to generate an item with extremeness level three, the value was chosen from the interval .20 - .29. The extremenesses for each of the four items that were generated as medium items was chosen from levels 4, 5, 6, and 7. For the fifth item, ten simulation conditions were used, so that its extremeness was chosen from a different level in each condition.

When there were 3, 4 or 5 categories of response, ten levels of extremeness were also defined using specific threshold values for each situation. These ten levels were used as explained in the previous paragraph.

The communality of the items was also manipulated in order to obtain a set of five items with known consistency/discrimination levels in the population. The four items that were generated as medium items were related to four levels of communality each: very low (.30 –.34), low (.35 –.39), regular (.40 –.44), and medium (.45–49). The communality of each of these four items was randomly chosen from one of these four intervals, respectively. For the fifth item, ten simulation conditions were used, so its communality was chosen from a different interval in each condition: the first interval was .00–.09, the second .10–.19, and so on until the tenth interval, which was .90–.99.

A unidimensional loading matrix was related to the communalities at hand, and Monte Carlo simulation used to obtain a sample *N* = 200 of continuous responses to the five items. Then items were discretized using the threshold values, the Polychoric inter-item correlation matrix was computed and the following analyses were carried out. We focused on the outcomes related to the fifth item:

1. We checked if the correlation matrix was positive definite. When it was not positive definite, no further analyses were computed for this correlation matrix.
2. We computed the estimated communality based on the multiple correlation. If a Heywood case was detected, no further analyses were computed for this correlation matrix.
3. We computed MSA for the fifth item. When the value was lower than the MSA threshold, no further analyses were computed for this correlation matrix.
4. We computed the bias of the sample loading matrix compared to the one expected in the population. If the bias was larger than .80, no further analyses were computed for this correlation matrix.
5. We computed the sampling error variance, and if the value was larger than .85, no further analyses were computed for this correlation matrix.
6. We recorded the consistency/discrimination and extremeness values of the fifth item, and the set of five items at hand was considered to be suitable for FA.

The set of five items was also assessed by linear FA: in this case, the correlation matrix analyzed was the Pearson correlation, and the same analyses were again computed. Each correlation matrix was analyzed using the Unweighted Least Squares approach.

In the simulation study 10 levels of consistency, and 10 levels of extremeness were manipulated. There were 4 graded responses. The study was replicated 1,000 times, and the 10 × 10 × 4 × 1,000 = 400,000 samples were analyzed.

### 2.3. Results of simulation study

The percentage of problematic situations detected in the simulation study is shown in Table 2. As can be observed, the Non-linear UVA graded model had most difficulties when there were two response categories. In addition, the most frequent problems were unstable parameter estimates and non-information, noisy items (i.e., MSA lower than .50). As the number of categories increased, the percentage of problematic situations decreased. It is interesting to note that when the set of items (which were ordinal) were analyzed using the liner factor model, problematic situations become less frequent, and the most observed situation was non-information, noisy items. Heywood cases were not observed because the communality estimate that we used (based on multiple correlation) tends to underestimate communalities. We decided to keep using it because it does not depend on a factor extraction algorithm and can be obtained before FA is carried out. Table 2 shows the percentage of problems observed in the simulation study. Most of the difficulties appeared related to the graded model, and specially related to the two response categories (i.e., binary items). As far as the type of difficulties, unstable parameter estimates and non-information, noisy items were the most frequent ones.

**Table 2:** Percentage of problems observed in the simulation study.

Potential problems	Graded model				Linear model			
	Response categories				Response categories			
	2	3	4	5	2	3	4	5
Non positive definite	1.66	0.79	0.14	0.01	4.00	2.07	0.06	0.01
Heywood case	0.00	0.00	0.00	0.00	0.00	0.00	0.00	0.00
Non-information, noisy items	23.98	15.85	4.85	4.41	8.53	6.96	2.71	2.47
Biased loading estimates	10.17	1.89	0.21	0.01	0.01	0.00	0.00	0.00
Unstable parameter estimates	45.76	40.23	34.22	21.51	0.13	1.57	2.14	3.58

**Fig 2:**
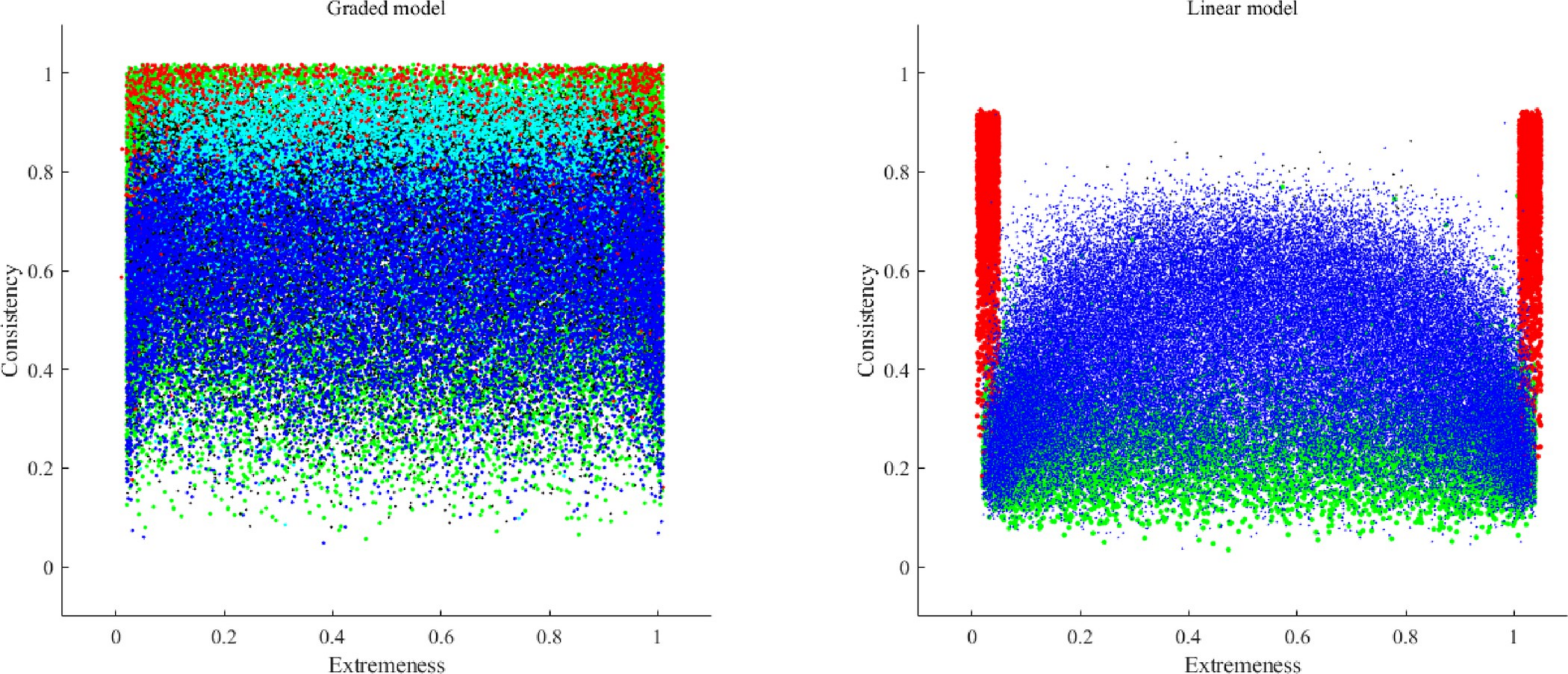
Graphic outcomes of the simulation study for two response categories.

**Fig 3:**
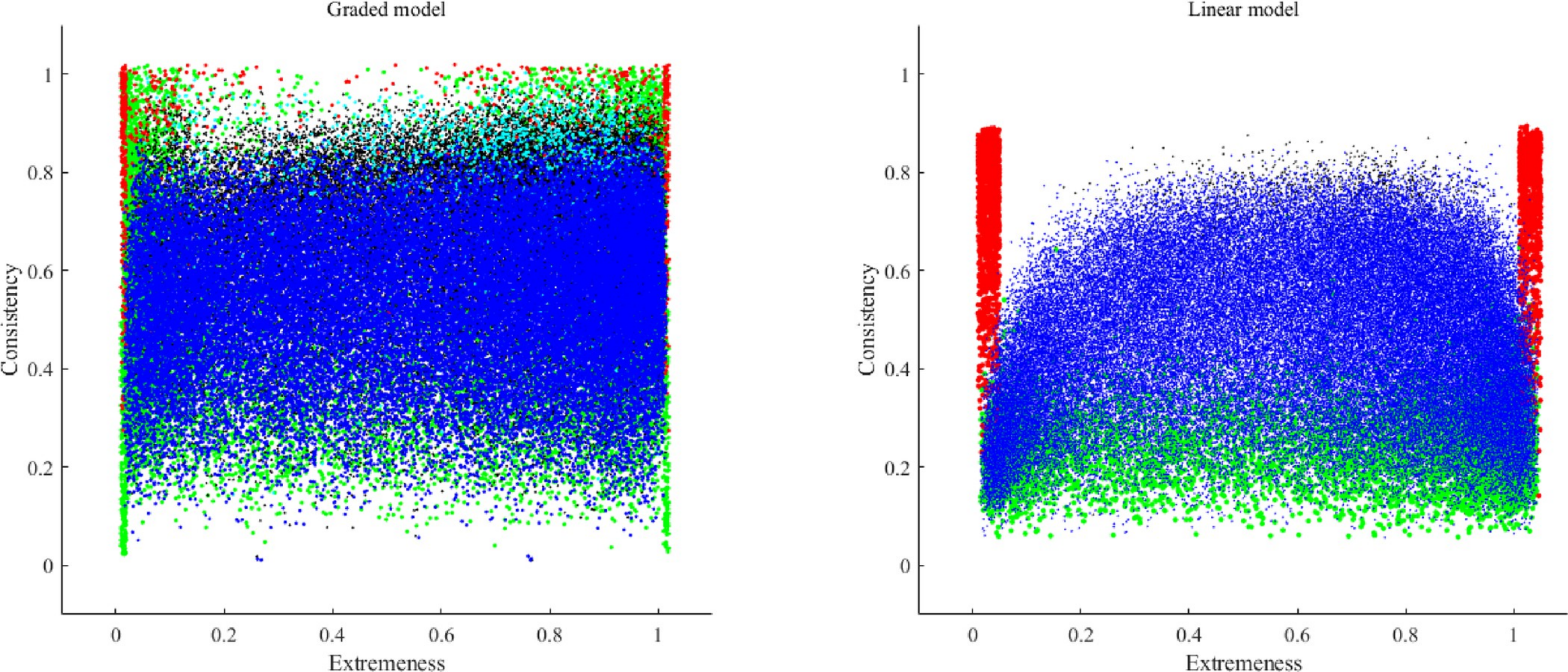
Graphic outcomes of the simulation study for three response categories.

**Fig 4:**
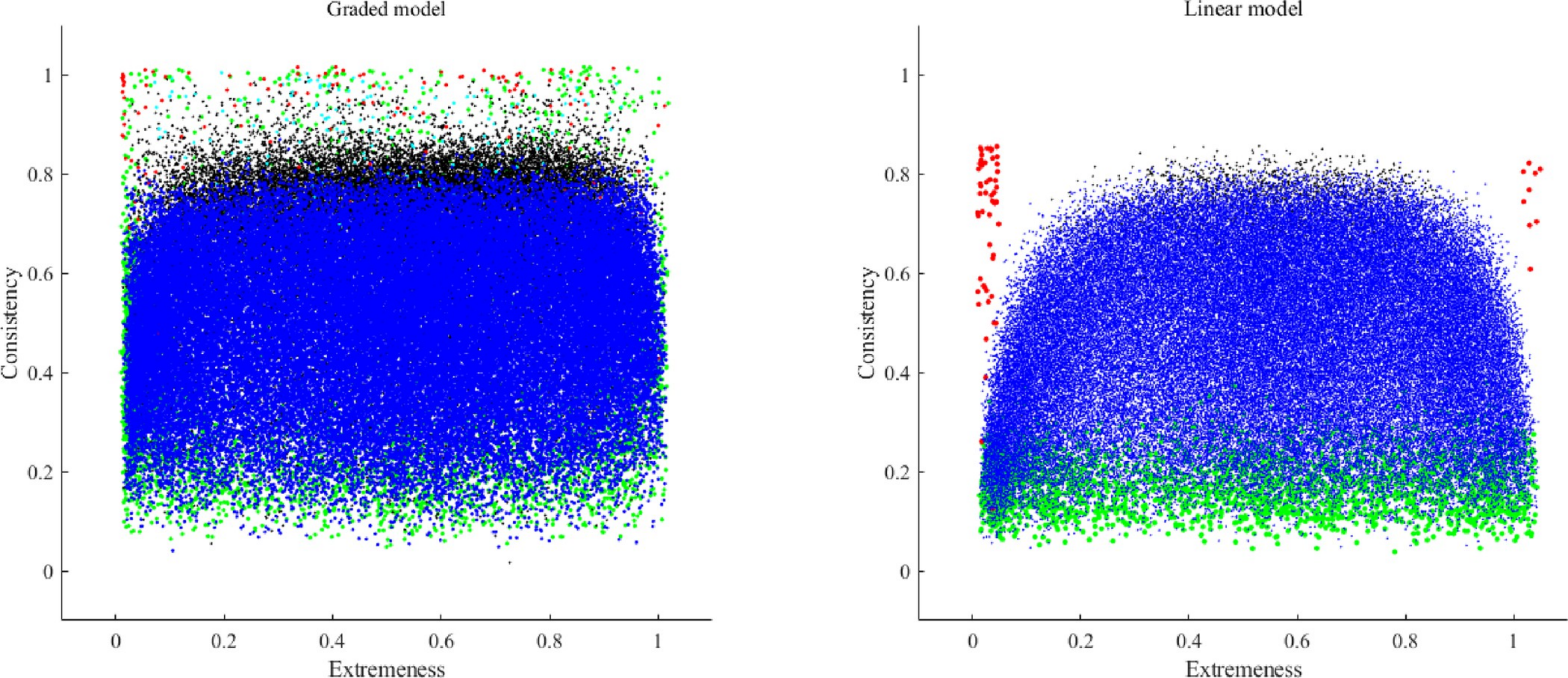
Graphic outcomes of the simulation study for four response categories.

**Fig 5:**
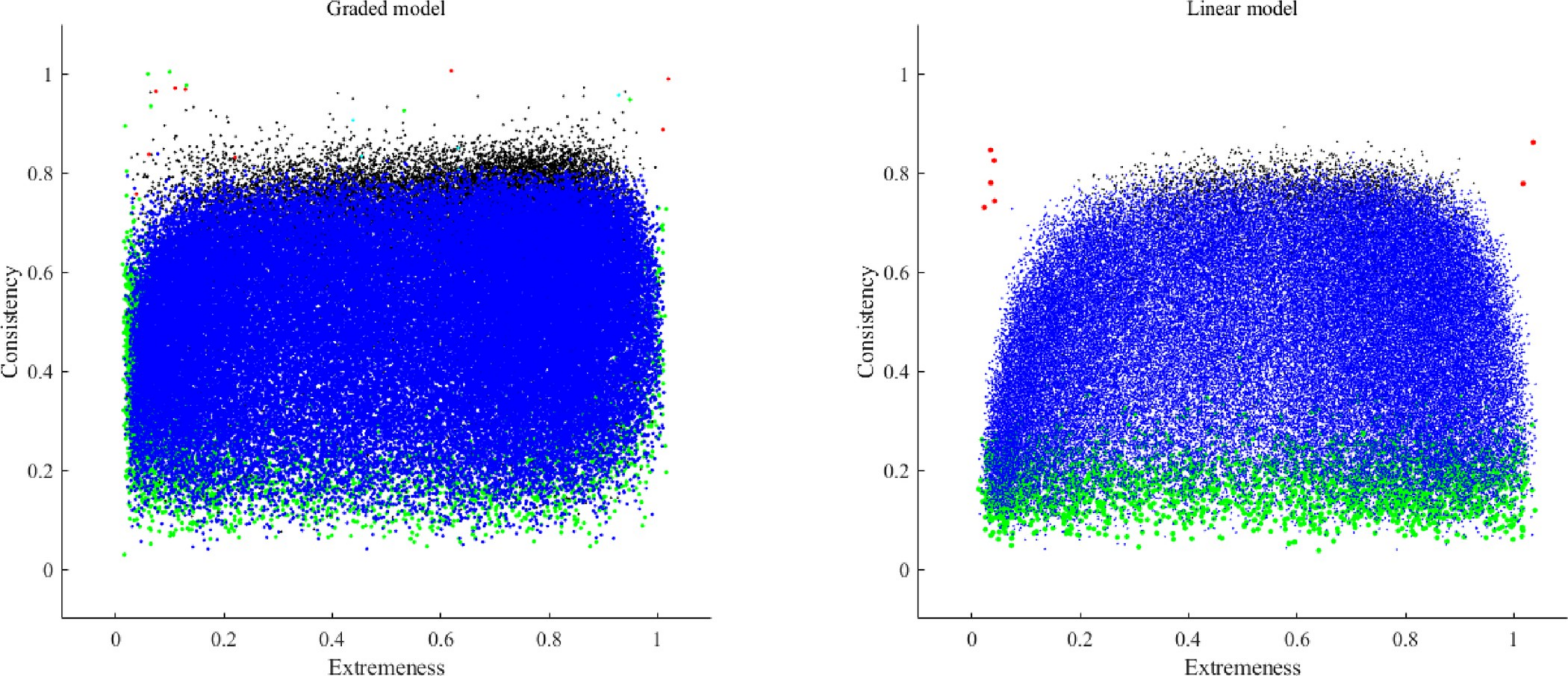
Graphic outcomes of the simulation study for five response categories.

Figs 2 to 5 show the consistency and the extremeness estimates of the fifth item in each dataset of the simulation study (one figure for each number of categories of response). Each item is represented using the following color code:

- Red: The correlation matrix turned out to be non-positive definite. In this case, the consistency is based on the population parameter, because the sample estimate could not be computed.
- Green: the MSA of the fifth items was lower than .50, and was considered to be a non-information, noisy item.
- Cian: the loading value estimates were biased in comparison to the population parameters.
- Black: the sample parameter estimates were unstable.
- Blue: no problem was detected for the set of items at hand.

### 2.4. Conclusions of simulation study

The inspection of the outcomes in Figs 2 to 5 helped us to define the initial boundaries of the pool. Even though there are some differences between the linear and the non-linear model, for the moment we prefer to propose a common region for both, until more information is available. The boundaries are: extremeness values between .10 and .90, and consistency values between .20 and .75.

**Fig 6:**
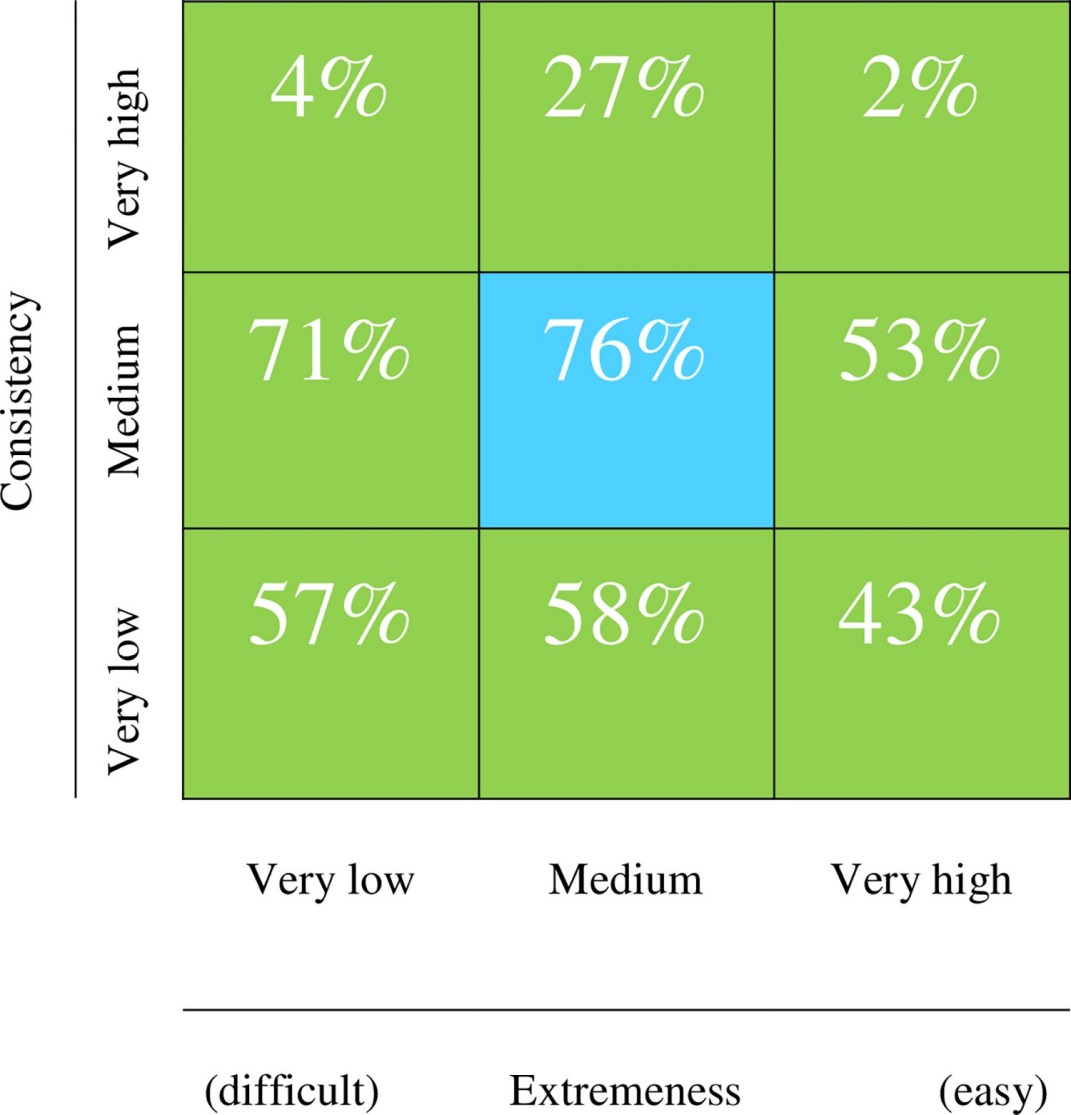
Averaged percentage of non-problematic items in each region of Gulliksen’s plot in the simulation study.

In order to report the utility of the boundaries that we defined, Fig 6 represents the different regions of Gulliksen’s pool already presented in Fig 1. The figure presents the average percentage of non-problematic items observed in the simulation study.

In general terms the overall results in Fig 6 agree with expectations. As expected, the central region is the one with least risk of problems. The bottom row does not have such a high proportion of problematic items, but the items in this row are expected to be uninformative and noisy (i.e. even when they do not greatly distort the structure, they are ineffective as measures). Finally, the worst expectations are in the top row, particularly at the ends, which suggests that keeping extreme, highly discriminating items in future phases of the analysis process is asking for trouble.

## 3. Advanced new proposals

### 3.1. An IRT re-formulation of the item efficiency index

Once an appropriateness region has been defined, it is desirable to provide further information about the amount of expected effectiveness for the items that it is recommended to keep. In principle, indices of item effectiveness that combine location and discrimination have already been proposed both within CTT [12] and within unidimensional IRT [30, 36]. In the present, possibly multidimensional, context, however, effectiveness must be ascertained without knowing the factorial composition of the item. So, what is to be assessed is, at best, the potential or prior capability of the item to provide accurate measurement. Furthermore, this type of assessment must be based on marginal (not conditional) properties, because not even the dimensionality of the item set is known.

In IRT, the most common measure of item effectiveness is the amount of information that the item response provides at different trait (or combination of trait) levels [30]. In particular, for the multidimensional 2PM the amount of information across the ‘optimal’ combination of traits is given by Eq 5.17 in [37],

$$I_{j}(\theta )=\left(\sum_{j}a^{2_{j}}\right)P_{j}(\theta )\left(1-P_{j}(\theta )\right)$$ (1)

The first term in (1) is the square of the multidimensional discrimination of item *j* [37]. Now, if, for the sake of simplicity, we use expression (1) and take the average of the amount of information across the trait levels, we obtain that the expected value can be approximated by

$$E_{\theta }(I_{j}(\theta ))≃\left(\sum_{j}a^{2_{j}}\right)P_{j}(1-P_{j})$$ (2)

where *P*_*j*_ is the marginal expectation of the *j* item score (i.e. the index of the extremeness we are considering in this article). The approximation in (2) can be considered as reasonably accurate in practice [30] (p. 45).

We turn now to the index we propose. The idea is first to use the item communality *h*^*2*^ as a ‘prior’ measure of squared multidimensional item discrimination, and then propose the index (2) based on the marginal expectations or estimated item extremeness. We call our index “Prior Item Efficiency” (PIE)

$${\mathrm{PIE}}_{j}=h_{j}^{2}P_{j}(1-P_{j})$$ (3)

Conceptually, PIE can be (approximately) interpreted as the potential or prior amount of item information (and so effectiveness) that this item can provide across all the hypothetical (trait) factor levels.

So far, our proposal has been derived from information measures that were intended for binary items. Therefore, it makes sense to ask to what extent PIE will be appropriate for graded-response or even more continuous items [38]. We believe that it will continue to be so. First, we are using a proportion-score metric that applies to any item format. Second, even though an item response can be made far more continuous than binary, it is still bounded. So, at the response scale endpoints there must be reduced variability and, therefore, a loss of information [16, 36, 38].

Inspection of (3) shows at once that the most effective items are those that are as discriminating as possible and not too extreme. However, what we propose here is a ceiling or an upper threshold for discrimination/consistency above which redundancy and instability rather than efficiency can be expected. To address this concern what we propose is to use a normed index that would provide the relative item efficiency with respect to the maximum efficiency that can be achieved given the upper ceiling proposed for *h*. If we denote this ceiling by *h*_*uppe*r_, the maximum PIE that can be attained is

$${\mathrm{PIE}}_{j}(\max)=h_{\mathrm{upper}}^{2}0.25$$ (4)

And the relative PIE (R-PIE) is given by:

$$R-{\mathrm{PIE}}_{j}=\frac{{\mathrm{PIE}}_{j}}{{\mathrm{PIE}}_{j}(\max)}=\frac{h_{j}^{2}P_{j}(1-P_{j})}{h_{\mathrm{upper}}^{2}0.25}$$ (5)

**Fig 7:**
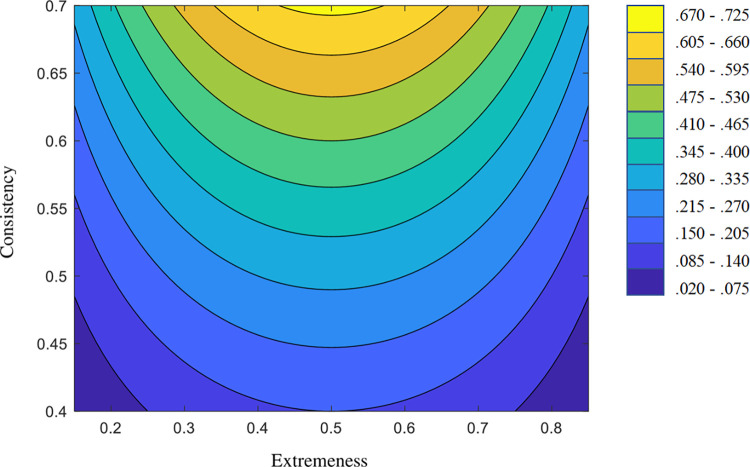
Contour plot for the relative prior item efficiency.

Fig 7 displays the contour plot of the R-PIE for the basic boundaries we proposed above. The interpretation of the contour plot in Fig 7 is quite clear: First, a certain amount of efficiency only starts to be obtained when there is a minimum amount of discriminating power/consistency. Second, as expected [12], efficiency increases as the item location approaches the 0.5 midpoint scaling level, and decreases as the item becomes more extreme in any direction.

### 3.2. A general proposal

In this section we propose a simple two-step procedure designed **as an aid** for obtaining a ‘cleaned’ efficient item pool that can be calibrated using FIA.

Step 1. From the initial item pool, examine the items that (a) fall outside the boundaries of the appropriateness region (for the model to be fitted) and (b) have MSA values below the proposed 0.5 threshold value. As discussed above, the items flagged in (a) are those considered to be potentially problematic and /or ineffective for various reasons. The items flagged in (b) are non-informative and behave almost at random in relation to the remaining items in the pool. After careful inspection of the item contents, decide which items are to be discarded.

Step 2. If there are enough surviving items in the trimmed pool after step 1, sort the non-rejected items in terms of their R-PIE estimates and keep the most effective items until the set is large enough to be used as input for fitting the FIA solutions.

### 3.3. Software implementation

We implemented G-pool in two existing statistical programs, and made it available at the web page of our university (http://www.psicologia.urv.cat/en/tools/). The code also computes the R-PIE. The code files developed are:

- The R script “GulliksenPool.r”. This script uses only native functions in R, so no packages need to be downloaded. To use it, researchers have to store participants’ responses in a text file, update the name of the input file, and execute the script. The number of bootstrap samples, the confidence interval, and the threshold values that define the pool can also be configured.
- The SPSS script “GulliksenPool.sps”. Again, to use this script, researchers must have participants’ responses in a SPSS data file, and execute the script. The same parameters as the R script can be configured.

Finally, we implemented the full item selection proposal (see below) in our program to compute FA. It can be downloaded free from the site http://www.psicologia.urv.cat/media/upload/domain_2082/arxius/Utilitats/factor/index.html. The computing is offered as a “PreFactor” method. To help the researcher to use PreFactor in FACTOR, a video tutorial is also available on the website.

## 4. Discussion

This article proposes and implements a comprehensive approach for use in the earlier stages of item selection. The approach is formulated from the general domain of EFA-based item analysis but is comprehensive because it is designed to work under a variety of psychometric models: CTT, FA and IRT. At its most basic level, the G-Pool procedure aims to flag potentially problematic items before any specific FA solution with a prescribed number of factors is fitted to the data. Next, the basic procedure is used in combination with an existing related procedure, robust-MSA, so as to obtain more information about which items are expected to be ineffective or problematic. Finally, a prior efficiency analysis is undertaken to help optimize the process of preliminary item selection if enough items are available.

The main results obtained here from the use of G-Pool agree generally with existing knowledge in the FIA literature. In particular, it is worth to highlight three of them. First, as expected, the most problematic items are those that are both extreme and highly discriminating [1, 3–4, 6, 38, 39]. Second, problems are minimized (and the model generally performs better) the more continuous the item format is [4, 6]. Third, the simple linear model tends to be more robust than its non-linear categorical-variable counterpart [4, 6, 16, 24].

The consistent results mentioned above can serve as a basis for making recommendations, but cannot be used uncritically. In fact, and in our view, the most important problem of what we propose here is the procedure being used in a “blind” automatic form. Thus, if the type and conditions of the test allows so, it is sensible advice to recommend the use of a graded format with a sizable number of categories in order to avoid or minimize problems in the FIA. However, in spite of this format being more convenient for the functioning of the model, the test might require in some cases the use of the simpler binary format (for example, for respondents with low cultural or motivational levels). Also, the graded format with a large number of categories is more prone to lead to bias related to the use of the response scale (e.g. extreme responding).

Things are more complex for the extremeness-discrimination issue. There is no problem about what to do with uninformative inefficient items. However, high discriminations are different. They might reflect good item quality, in which case, keeping these items might outweigh the possible risks. However, they may also reflect (a) redundancy or shared specificity or (b) very high discrimination at a single point and near zero discrimination in the rest of the range (e.g. a near-Guttman item). The latter case is discussed below, and, as for the former, the most convenient action may be to remove the redundant items. With regards to extremeness, finally, our experience suggests that item locations are seldom studied in FIA. However, as demonstrated here, they are highly relevant. So, it should be assessed whether the extremeness reflects a need of the study or is a design flaw. To sum up, there is nothing that exempts the researcher from thinking and deciding critically.

As discussed above, the present proposal is expected to be particularly useful when the conditions that lead to a trouble-free FIA are also the most convenient for the type of test and measurement aims, a scenario that is submitted to be the most common in applications. If the test requires “extreme” and highly discriminative, near-Guttman items [34], the proposal continues to be valid (these items are problematic for FIA regardless of the aims of the test) but the decisions become more complex. In the limiting case in which most of the items have to be of this type, then the FIA is probably not the most appropriate model and alternative models should be considered [13, 34].

Our proposal starts from an existing idea (the basis bivariate plot) and uses many known results and concepts that belong to the CTT, IRT and FA domains. Even so, we believe that it contains new developments and that it makes a contribution in many regards. As for new developments, we shall consider four. First, the regions of appropriateness we propose are obtained by using extensive simulation, and taking into account the two FA models that are available for item analysis: linear and UVA-nonlinear. Second, the regions can be defined in the factor-analytic and IRT metric. Third, a robust approach that takes into account the sampling variability of the item coordinates is used to flag the offending items. Finally, the absolute and relative indices of prior item efficiency are, to the best of our knowledge, new proposals.

As for limitations, we have checked how the proposal works only at the calibration stage by considering the impact that the problematic items have at the structural level. However, the amount of item efficiency is ultimately related to the quality of the score estimates derived from the structural FA solution and this point has not been studied. In principle, what is expected is that discarding the most extreme items would prevent end (floor and ceiling) effects from appearing, thus increasing the effective differentiation of individuals. On the other hand, selecting items with appropriate consistency/discrimination is expected to lead to more accurate score estimates. These issues warrant further intensive research that was beyond the scope of this article.

In spite of its limitations, we believe that this proposal has wide applicability and will be useful for the practitioner. At the substantive level, its rationale is simple and clear, and it can save the researcher a lot of trouble. At the instrumental level, our approach is implemented in several statistical packages. Furthermore, the example that we provide on our website in R should help other researchers to implement it in other packages.
